# Evaluation of health-related quality of life via the Computer-Based Health Evaluation System (CHES) for Japanese metastatic breast cancer patients: a single-center pilot study

**DOI:** 10.1007/s12282-018-0905-1

**Published:** 2018-09-07

**Authors:** Yuichiro Kikawa, Yukimasa Hatachi, Gerhard Rumpold, Mariko Tokiwa, Sayaka Takebe, Takatsugu Ogata, Hironaga Satake, Hironori Kato, Akihito Tsuji, Hisateru Yasui, Bernhard Holzner

**Affiliations:** 1 Department of Breast Surgery, Kobe City Medical Center General Hospital, 2-2-1, Minatojima Minamimachi, Chuo-ku, Kobe, Hyogo 650-0047 Japan; 2 Department of Medical Oncology, Kobe City Medical Center General Hospital, Kobe, Japan; 3 Department of Medical Oncology, Kagawa University Faculty of Medicine, Miki, Japan; 4 Department of Psychiatry, Psychotherapy and Psychosomatic Medicine, Medical University of Innsbruck, Innsbruck, Austria

**Keywords:** Breast cancer, Quality of life, Electronic patient-reported outcome, CHES

## Abstract

**Background:**

The main purposes of metastatic breast cancer (MBC) treatment are to prolong survival and maintain health-related quality of life (HRQOL). Compliance with the HRQOL assessment can be poor, particularly among patients who receive long-term treatment. One possible solution to overcoming this problem is to engage in real-time home monitoring by having patients report outcomes on their personal electronic devices. The objective of this study was to investigate compliance with HRQOL monitoring from home among MBC patients using the Computer-Based Health Evaluation System (CHES) to collect patient data.

**Methods:**

Sixteen MBC patients who received outpatient chemotherapy or endocrine therapy, both with and without targeted therapy, were recruited. One eligibility criterion was the availability of a personal electronic device with access to the Internet. Patients were asked to enter HRQOL ratings from their personal electronic devices via CHES once every week for 12 weeks. The European Organization for Research and Treatment of Cancer (EORTC) Quality of Life Questionnaire-Core 30 (QLQ C30) was used to evaluate HRQOL. The outcome examined was the questionnaire collection rate.

**Results:**

Six patients (37.5%) were treated with chemotherapy only, one (6.2%) with endocrine therapy only, three (18.8%) with a combination of chemotherapy and targeted therapy, and six (37.5%) with a combination of endocrine and targeted therapy. Median questionnaire collection rate for the total of 12 weeks was 84.6% (interquartile range 44.3–100). The reasons for missing data were worsening of disease, forgetting, and device malfunction.

**Conclusions:**

Compliance with electronic HRQOL data collection in this cohort was acceptable, considering the general ideal collection rate of 70–80%. We are conducting a prospective study to determine whether the use of CHES to input electronic real-time feedback of HRQOL ratings improves patients’ overall HRQOL.

## Introduction

Metastatic breast cancer (MBC) is an incurable disease. The main purposes of treatment are to prolong survival and maintain health-related quality of life (HRQOL). There are two crucial problems involved in assessing HRQOL. First, physician ratings frequently underestimate the patient’s real symptom burden [1]. Second, although an ideal questionnaire collection rate is 70–80% in general, compliance with the assessment of HRQOL among patients diagnosed with MBC can be poor [2], particularly among those receiving long-term treatment. The electronic data capture of patient-reported outcomes (ePRO) and real-time home monitoring by means of the patient’s tablet computer, mobile phone, or personal computer may overcome these two barriers to HRQOL reporting. However, despite the fact that Japan is a developed country, the use of ePROs is not widespread. This study implemented a clinical application of ePRO for breast cancer patients. The objective of this study was to investigate how the use of the Computer-Based Health Evaluation System (CHES) impacts the rate of home HRQOL monitoring compliance. This study was registered with the University Hospital Medical Information Network (UMIN) Clinical Trials Registry managed by the National University Hospital Council of Japan (UMIN 000023250).

## Patients and methods

**Fig. 1:**
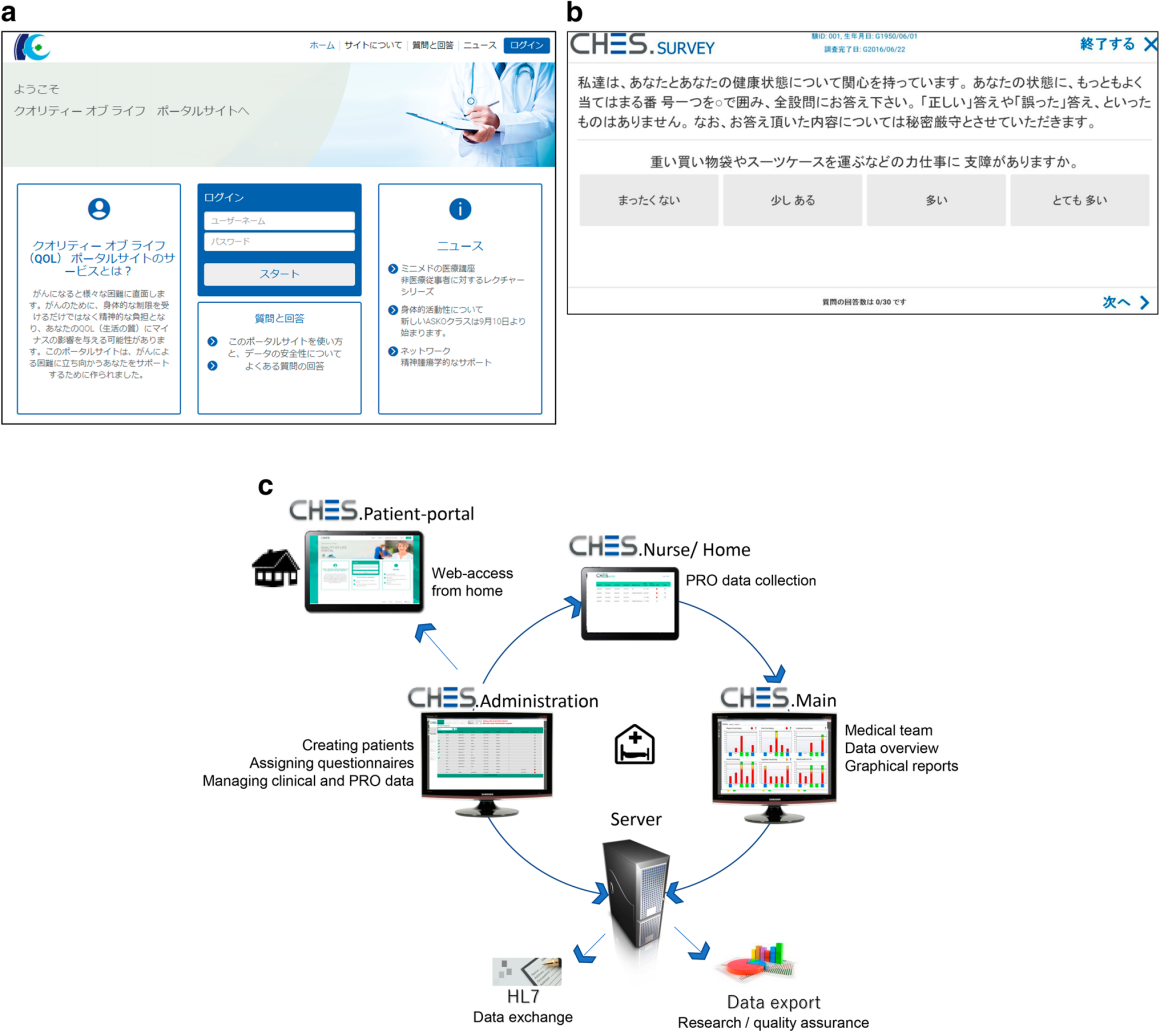
**a** Screen of the patient portal site. After patients enter their anonymized ID and password, they move on to the screen of the QOL questionnaire. *QOL* quality of life. **b** Example of the Japanese version of the QOL questionnaire. *QOL* quality of life. **c** Data flow diagram of HRQOL ratings inputted into the CHES, developed by EORTC. *HRQOL* health-related quality of life, *CHES* Computer-Based Health Evaluation System, *EORTC* European Organization for Research and Treatment of Cancer, *PRO* patient-reported outcome, *HL7* Health Level Seven

This single-center pilot study was approved by the Institutional Review Board. CHES is a platform that electronically collects patient questionnaires developed by the European Organization for Research and Treatment of Cancer (EORTC) QOL group [3]. An anonymized ID and a password are assigned to each registered patient. Participants then use their personal electronic devices to login to CHES, view the patient portal site (Fig. 1a), and complete the QOL questionnaires (Fig. 1b). The obtained data are stored in a secure server. Medical staff can access the QOL data at any time and display the longitudinal data on a graphical screen (Fig. 1c). In this study, we used a Japanese version of a questionnaire jointly developed with the EORTC QOL group.

Between November 2016 and January 2017, 16 consecutive MBC patients who received outpatient chemotherapy or endocrine therapy, with or without molecular targeted therapy, at Kobe City Medical Center General Hospital were recruited to participate in this study. The availability of an electronic device with access to the Internet was required in order for each participant to be eligible to participate in the study. There were no other eligibility criteria. Participants provided written informed consent. Patients were asked to utilize CHES to enter values of HRQOL ratings from their personal devices once every week for 12 weeks. The EORTC Quality of Life Questionnaire-Core 30 (EORTC QLQ C30) was used to evaluate HRQOL. EORTC QLQ C30 is a 30-item questionnaire for the assessment of HRQOL among various types of cancer patients, and it is the most often used measurement tool for patient-reported outcomes (PRO) [4]. The percentage of HRQOL questionnaires completed in the CHES was the outcome measured. We defined an HRQOL questionnaire as having been completed if the patient answered at least one question. We also investigated the reason for missing data from patients with a compliance rate below 80%, which we defined as poor compliance.

## Results

The patients’ background information is shown in Table 1. Participants ranged in age from 38 to 70 years, with a median age of 58 years. At the start of the survey, nine patients (56.2%) had an Eastern Cooperative Oncology Group performance status of 0, while five (31.3%) and two (12.5%) had a performance status of 1 and 2, respectively. Six patients (37.5%) were treated with chemotherapy only, one (6.2%) with endocrine therapy only, three (18.8%) with a combination of chemotherapy and targeted therapy, and six (37.5%) with a combination of endocrine and targeted therapy.

**Table 1:** Patient background and demographics

	Median (range)	*n* (%)
Age (in years)	58 (38–70)	
ECOG performance status		
0		9 (56.2)
1		5 (31.3)
2		2 (12.5)
Treatment regimen		
Chemotherapy only		6 (37.5)
Chemotherapy + targeted therapy		3 (18.8)
Endocrine therapy only		1 (6.2)
Endocrine + targeted therapy		6 (37.5)
Highest level of education		
High school		3 (18.8)
Career college		3 (18.8)
Junior college		4 (25.0)
University level or above		4 (25.0)
Unknown		2 (12.5)
Employment status		
Full-time		3 (18.8)
Part-time		3 (18.8)
Homemaker		6 (37.5)
Unemployed		2 (12.5)
Retired		2 (12.5)
Marital status		
Single		5 (31.3)
Married/partner		10 (62.5)
Separated/divorced		1 (6.3)

*ECOG* Eastern Cooperative Oncology Group

**Fig. 2:**
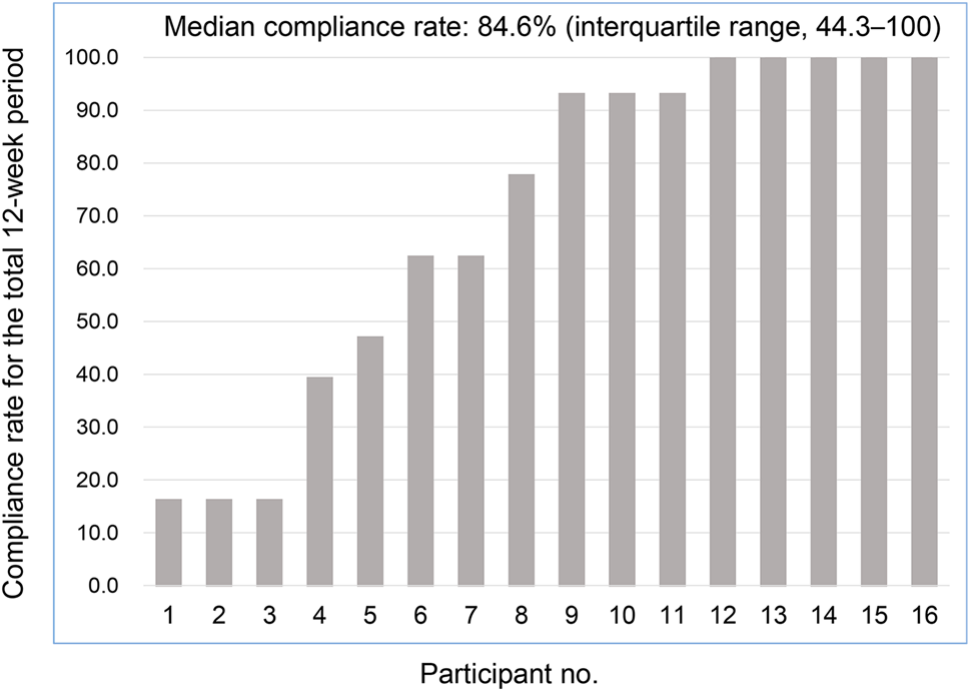
Questionnaire collection rate. *HRQOL* health-related quality of life, *no*. number

Ten patients (62.5%) accessed the CHES on personal computers, four (25.0%) on smartphones, and two (12.5%) on tablets. Fifteen patients (93.8%) accessed the CHES and rated their own HRQOL values, while one patient (6.3%) required family assistance to input these data (Table 2). Median questionnaire collection rate for the total of 12 weeks was 84.6% (interquartile range 44.3–100) (Fig. 2). The reasons for missing data among eight patients with collection rates of 80% or less were worsening of disease for four patients (50.0%), forgetting for three (37.5%), and device malfunction for one (12.5%).

**Table 2:** Input device and questionnaire contributor

	*n* (%)
Input device	
Personal computer	10 (62.5)
Smartphone	4 (25.0)
Tablet	2 (12.5)
Questionnaire contributor	
Patient	15 (93.8)
Family	1 (6.3)

## Discussion

Our pilot study implemented, for the first time in Asia, the CHES developed by EORTC to electronically evaluate HRQOL among a small number of participants. The median compliance rate for the questionnaire input was 84.6%. Generally, the ideal rate of collection is considered to be 70–80% in order to conduct an appropriate QOL evaluation in a clinical trial. Based on this, our findings demonstrate the feasibility of using CHES although there is no clear threshold of collection rate regarding the monitoring of patients using ePRO. However, the range of compliance was extensive and polarized between patients with high and poor compliance rates. The main reasons why patients with poor compliance did not enter answers were the worsening of disease and forgetting. Because the study subjects were MBC patients, their disease progression was inevitable. Meanwhile, e-mail reminders may be needed for patients who have trouble remembering to answer questionnaires. Future studies may explore ways to improve patient participation and reduce missing data.

This study did have some limitations. Namely, there was a small sample size, and participants were only eligible to participate if they had access to the Internet. However, a survey on Japanese communication usage trends conducted by the Ministry of Internal Affairs and Communications [5] found that the Internet use rate of individuals in Japan in 2013 was 82.8%, and that it has increased every year among all age groups. Consequently, the frequency of ePRO use is expected to expand further within a few years.

Applying ePRO to daily clinical practice for cancer patients presents several challenges [6]. Basch et al. showed in a randomized trial that symptom monitoring at home using ePRO contributes to the improvement of HRQOL and the prolongation of survival for solid cancer patients undergoing outpatient chemotherapy [7]. Denis et al. demonstrated similar results among lung cancer patients [8]; therefore, Internet-based HRQOL monitoring may be an effective way of prolonging survival for cancer patients. However, it is uncertain whether similar results would be obtained in Japan since, compared to other developed countries, Japanese patients have increased access to hospitals thanks to the national health insurance system. That is, good accessibility to medical facilities, regardless of rate of HRQOL monitoring, may prolong survival rates and reduce the survival benefit of using ePRO. Therefore, we are conducting prospective research to explore the usefulness of CHES for solid cancer patients in Japan where access to hospital facilities is unlimited.

In conclusion, compliance with electronic HRQOL data collection in this cohort was acceptable. In the future, we will continue to explore whether using CHES to electronically input real-time HRQOL ratings improves MBC patients’ overall HRQOL and rate of survival in Japan (UMIN000029663).
